# In Vivo Relevance of a Biphasic In Vitro Dissolution Test for the Immediate Release Tablet Formulations of Lamotrigine

**DOI:** 10.3390/pharmaceutics15102474

**Published:** 2023-10-17

**Authors:** Tuba Incecayir, Muhammed Enes Demir

**Affiliations:** Department of Pharmaceutical Technology, Faculty of Pharmacy, Gazi University, 06330 Ankara, Turkey

**Keywords:** biphasic in vitro dissolution, in vitro-in vivo correlations (IVIVC), lamotrigine

## Abstract

Biphasic in vitro dissolution testing is an attractive approach to reflect on the interplay between drug dissolution and absorption for predicting the bioperformance of drug products. The purpose of this study was to investigate the in vivo relevance of a biphasic dissolution test for the immediate release (IR) formulations of a Biopharmaceutics Classification System (BCS) Class II drug, lamotrigine (LTG). The biphasic dissolution test was performed using USP apparatus II with the dual paddle modification. A level A in vitro-in vivo correlation (IVIVC) was constructed between the in vitro partition into the octanol and absorption data of the reference product. A good relation between in vitro data and absorption was obtained (r^2^ = 0.881). The one-compartment open model was introduced to predict the human plasma profiles of the test product. The generic product was found to be bioequivalent to the original product in terms of 80–125% bioequivalence (BE) criteria (85.9–107% for the area under the plasma concentration curve (*AUC*) and 82.7–97.6% for the peak plasma concentration (*C_max_*) with a 90% confidence interval (CI)). Overall, it was revealed that the biphasic dissolution test offers a promising ability to estimate the in vivo performance of IR formulations of LTG, providing considerable time and cost savings in the development of generic drug products.

## 1. Introduction

In vitro dissolution testing is an essential step in drug product development and a routine manufacturing process for quality control purposes, as well as in the establishment of in vitro-in vivo correlations (IVIVC) and the prediction of the in vivo behavior of drug formulations [1]. Compendial dissolution tests, which aim for sink conditions, are usually carried out in buffer solutions and are adequately used for quality control. However, the ability of a dissolution test to perform in vivo prediction mainly depends on its in vivo relevance. In pharmaceutical research, there is a continuous search for physiologically relevant dissolution media, conditions, and in vitro tools to predict the in vivo behavior of oral formulations in the human gastrointestinal (GI) tract and understand the effects that the main processes on oral bioavailability (BA) of drugs in humans [2,3,4,5,6].

For poorly water-soluble drugs, drug dissolution mainly depends on the pH and dissolution of the medium composition (buffer type, capacity, and volume) [7]. Thus, maintaining sink conditions for the complete dissolution and characterization of these drugs can be challenging. Moreover, Biopharmaceutics Classification System (BCS) Class II drugs (low solubility-high permeability) that exhibit dissolution rate-limited absorption are considered to be good candidates for IVIVCs and develop in vivo predictive dissolution tests reflecting in vivo solubility, release, and absorption to forecast the in vivo behavior of their orally administered formulations [5,6,8].

One attractive approach is the use of biphasic dissolution systems to afford sink conditions and better reflect the interplay between drug dissolution and absorption to predict the in vivo behavior of formulations containing BCS Class II drugs. Biphasic systems depend on a dissolution medium comprising immiscible aqueous and organic phases [9]. In a biphasic dissolution system, drug partition into the organic solvent occurs, depending on the drug’s lipophilicity following initial dissolution in the aqueous phase. Moreover, the organic solvent can create a more realistic drug supersaturation in the buffer [10]. The organic phase (octanol) is considered to mimic the absorption of the drug through the intestine, maintaining sink conditions [11]. In biphasic dissolution test studies, the best organic solvent is considered to be octanol [12]. Therefore, it is commonly used for biphasic systems due to its suitable physicochemical properties, such as water insolubility (0.05 g/100 g water) and low density (specific gravity of 0.825 at 20 °C), permitting the ease of sampling and low volatility (boiling point at 195 °C); this prevents evaporation at 37 °C and keeps the upper phase volume constant [13,14].

Niebergall et al. were the first to investigate the partitioning rates of salicylic acid tablets in a vessel containing 250 mL of octanol and aqueous phases [15]. Similarly, using a biphasic system, Gibaldi and Feldman also determined the first-order dissolution rates of benzoic and salicylic acids [16]. The correlation between a biphasic system and in vivo results was first investigated for oral ibuprofen formulations by Stead et al., resulting in a promising IVIVC but requiring improvement [17]. Different system configurations for biphasic dissolution testing have been reported in the literature, including a USP apparatus II with dual paddle, a basket–paddle hybrid stirrer, and a USP apparatus IV coupled with a USP apparatus II [13,18,19]. Lately, a miniaturized system (miBIdi-pH, 50 and 15 mL of aqueous and organic phases, respectively) was developed to investigate the supersaturation, precipitation, and absorption kinetics of six BCS II model drugs (griseofulvin, ibuprofen, dipyridamole, telmisartan, fenofibrate, and itraconazole) [20,21]. In a recent study, small-scale biphasic systems were proposed to assess the intraluminal performance of poorly soluble, ionizable compounds in early drug development due to the limited quantities of drug substances available [22]. The latest studies on mathematical models to express the partitioning kinetics of a compound between aqueous (buffered solutions) and organic (octanol) phases and mass transport analysis on the partitioning kinetics of BCS II drugs (ibuprofen, nimesulide, and piroxicam) provides a deep understanding of biphasic testing [23,24]. In the last 50 years, studies have demonstrated that biphasic dissolution tests can be applied to different dosages, such as controlled-release formulations, soft gelatin capsules, tablet formulations, lipid-filled capsules, self-emulsifying drug delivery systems, nanoemulsions, and lipid-based solid dispersions. They have a promising potential to discriminate formulations, conduct IVIVCs, and predict in vivo behavior, maintaining sink conditions [18,25,26,27,28,29,30,31].

The purpose of the present study is to investigate the in vivo relevance of a biphasic dissolution test for the immediate release (IR) formulations of a BCS Class II drug, lamotrigine (LTG). This study describes the application of a biphasic dissolution test to establish an IVIVC for IR formulations (reference and test) containing 200 mg of LTG (CAS 84057-84-1) and investigates its relevance regarding in vivo absorption in humans. To the best of the authors’ knowledge, there is no study on biphasic dissolution testing for oral formulations of LTG in the literature.

LTG, a phenyltriazine class anticonvulsant, shows efficacy against partial and generalized epilepsies. It blocks voltage-sensitive sodium channels and inhibits the release of excitatory neurotransmitters [32]. As a BCS Class II drug, it is adequately absorbed from the GI tract with a BA approaching 98% and the peak plasma concentration (*C_max_*) occurring within about 3 h postdose [33,34]. The steady-state elimination half-life (t_1/2_) is 25 to 30 h in healthy young adults [35]. The total urinary recovery of the oral dose is 70%, mainly in the form of glucuronide conjugate [36].

Conventional and biphasic dissolution tests for bioequivalent IR formulations (reference and test) of LTG (200 mg) were evaluated in the present study. Previously reported plasma concentration–time curves after the oral administration of the LTG reference formulation in healthy volunteers were used to correlate these data with the results obtained from the biphasic dissolution test [37]. Based on this correlation, a model-dependent approach was introduced to estimate the in vivo performance of the IR product of LTGs in humans.

## 2. Materials and Methods

### 2.1. Materials

LTG (purity: 99.9%) was kindly provided by Sanovel Pharmaceuticals (Istanbul, Turkey). Sodium chloride, sodium hydroxide, hydrochloric acid, n-octanol, and potassium phosphate monobasic were supplied from Sigma-Aldrich (Steinheim, Germany). Two commercial IR (soluble and chewable) tablets of 200 mg of LTG were obtained from a local pharmacy. The commercial reference, A8158013, and test, 19143001, tablets were employed. The chemicals were of analytical reagent grade.

### 2.2. Single Phase In Vitro Dissolution Test

A conventional dissolution test of the drug products was conducted using a 708-DS Agilent dissolution apparatus (Agilent Technologies, Selangor, Malaysia) under sink and non-sink conditions. The commercial IR tablets were tested using USP dissolution apparatus II, with 900 mL of the dissolution medium (pH 1.2 HCl and pH 6.8 phosphate buffers (50 mM)) at 37 °C and the paddle rotating at 50 rpm. Withdrawn samples (5 mL) at predetermined times were filtered using a 0.45 µm Chromafil^®^CA45/25 syringe filter, and an equivalent amount of fresh medium was added into each vessel after sampling. The determination of LTG was performed using the spectrophotometric method. The tests were carried out in six replications. The cumulative percentage was dissolved (mean ± standard deviation (SD)) and plotted versus time.

### 2.3. Biphasic In Vitro Dissolution Test

**Figure 1:**
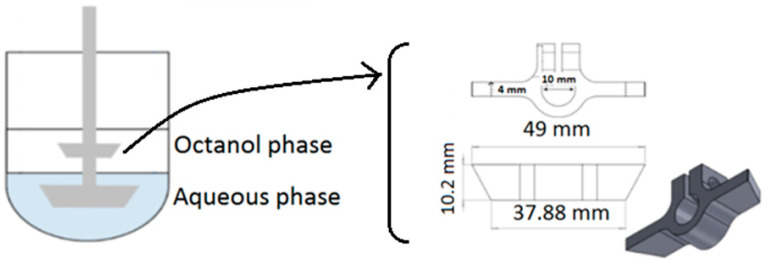
Schematic depiction of USP apparatus II with the additional paddle in the present study.

A biphasic dissolution test was carried out using paddle-modified USP apparatus II (Figure 1). An additional paddle (stainless steel, grade 316) was fixed above the compendial paddle. The phosphate buffer (pH 6.8, 50 mM, 300 mL) and octanol (200 mL) were used as the aqueous and organic phases of the dissolution media, respectively. The paddle speed was set to 50 rpm at 37 °C. The aqueous and organic phases were mutually saturated with stirring for 30 min prior to the dissolution run. The commercial IR tablets were introduced into the aqueous phase using a tube passed through the organic phase to avoid the tablet’s contact with the octanol. The volume of octanol (200 mL) was selected based on in vitro partitioning into octanol, as well as the saturation solubility (*C_S_*) of LTG into octanol (4.14 mg/mL) to provide the relative sink condition in 200 mL of octanol. The partitioning rate coefficient of LTG into octanol (*k_p_*) was calculated with linear regression using Equation (1) [24].

(1) $${\;F}_{o,\;t}=1-e^{-k_{p}t\;}$$

where *F*_o,*t*_ is the fraction of LTG in the organic medium, and *t* is time.

Increasing the organic phase from 200 to 300 mL did not affect the *k_p_* value of LTG (0.62 vs. 0.66 h^−1^), while the rotating speed of 75 rpm increased the *k_p_* value approximately two times (1.4 h^−1^) compared to 50 rpm in the present study. The effect of the rotating speed on the partitioning rate was also confirmed for dipyridamole by other researchers [21]. However, a paddle speed of 50 rpm was used to avoid undesirable mixing and turbulence, which occurred at the interface of the two phases at 75 rpm in the present study, as recommended by the others [24].

The additional paddle was put at the center of the octanol phase to provide sufficient stirring. The aqueous phase volume (300 mL) ensured that the USP II compendial paddle was entirely in the aqueous phase. Samples (5 mL) were withdrawn from the aqueous and organic phases at 15, 30, 45, 60, 90, 120, 180, and 240 min, respectively, and filtered using a 0.45 µm syringe filter (Chromafil^®^CA45/25, Macherey-Nagel GmbH and Co.KG, Dueren, Germany), and equivalent amounts of respective fresh media were added. Withdrawn samples were analyzed using the spectrophotometric method. The tests were performed in triplicate. The mean cumulative percent (mean ± SD) in the aqueous buffer and octanol was plotted as a function of time.

### 2.4. Assay

The samples were analyzed by spectrophotometry using a Cary 60 UV-Vis spectrophotometer (Agilent Technologies, Santa Clara, CA, USA). The absorbance of the samples in the two-dissolution media (pH 1.2 and pH 6.8) and in octanol were measured at 265, 305, and 310 nm, respectively. The concentration of LTG was determined by the calibration curves of each corresponding dissolution medium. Linearity was obtained in a calibration range of 7.5–35 μg/mL (r^2^ = 0.999). Accuracy ranged from 98.3% to 101%. The relative SD of within-day and between-day precision values was less than 1.0%. The limit of quantification (LOQ) and the limit of detection (LOD) values were 3.0 and 1.0 µg/mL in octanol, respectively. LOQ values were 1.2 and 0.9 µg/mL, and LOD values were 0.4 and 0.3 µg/mL at pH 1.2 and pH 6.8 in dissolution media, respectively.

### 2.5. In Vitro Data Analysis

Dose number (*D_O_*), defined as the mass divided by an uptake volume of 250 mL and the solubility of the drug, was calculated using Equation (2) [38]:

(2) DO=MOCS.VO

where *M_O_* is the highest dose (mg), *V_O_* is the initial gastric volume, and *C_S_* is the saturation solubility (mg/mL).

All data were shown as the mean ± SD. The similarity of drug products was determined using the *f_2_* similarity test [39]. *f_2_* values were calculated using Equation (3):

(3) $$f_{2}=50\;log\frac{100}{\sqrt{1+\frac{1}{n}\sum_{t=1}^{n}{\left(R_{t}-T_{t}\right)}^{2}}}$$

where *n* is the sample number, *R_t_* and *T_t_* are the cumulative percentages of the reference and test products dissolved at time point *t*, respectively. The calculated *f*_2_ > 50 points correspond to the similarity of the two profiles.

### 2.6. Prediction of In Vivo Plasma Profiles from the Correlation Between Absorption and In Vitro Partitioning Data

LTG’s fraction of dose-absorbed (*F_abs_*) values (%) was derived from the published data of the reference product in healthy volunteers to assess the in vivo relevance of the biphasic dissolution test [37]. *F_abs_* values were calculated from the plasma data of LTG using the Wagner–Nelson method (Equation (4)) [37,40].

(4) $$F_{abs}=\frac{k_{d}\int_{0}^{t}C\left(t\right)dt+C\left(t\right)}{k_{d}\int_{0}^{\infty }C\left(t\right)dt}$$

where *C*(*t*) is the plasma drug concentration (µg/mL) and *k_d_* is the elimination rate constant (h^−1^).

In vivo and in vitro data were compared using a point-to-point relationship between the calculated *F_abs_* and the fraction of LTG partitioned into the octanol at 15, 30, 60, 120, and 240 min for reference. The relationship between in vivo and in vitro data was investigated using linear regression. All calculations were carried out using Microsoft Excel 2013. Using the level A IVIVC, *F_abs_* values of each healthy subject were found for the test product. Each subject’s absorption rate constant (*k_a_*) values for the test were calculated using the Wagner–Nelson method. The one-compartment open model was used for pharmacokinetic (PK) analysis. Plasma concentrations for the test (*C_p_*) were predicted with Equation (5) using the *k_a_* values calculated by the Wagner–Nelson method, as well as the elimination rate constant (*k_d_* = 0.0279 ± 0.0123 h^−1^) and volume of distribution (*V_d_* = 72.0 ± 9.9 L) data specific to each subject for the reference. Thus, the plasma concentration profiles of LTG versus time for the test product were obtained for each healthy subject. The area under the plasma concentration curve (*AUC*_0→∞_) values were calculated from zero to infinity using the trapezoidal rule method.

(5) $$C_{p}=\frac{FF^{*}D}{V_{d}}\frac{k_{a}}{k_{a}\;-{\;k}_{d}}\left(e^{-k_{d}t}-e^{-k_{a}t}\right)$$

where *C_p_* is the drug concentration in the plasma (µg/mL), FF* is the bioavailability constant, *D* is the drug dose (µg), *V_d_* is the volume of distribution (mL), *k_a_* is the absorption rate constant (h^−1^), *k_d_* is the elimination rate constant (h^−1^), and *t* is time (h).

### 2.7. Bioequivalence (BE) Analysis

BA criteria (*C_max_* and *AUC*_0→∞_) were calculated. The bioequivalence (BE) of the generic versus original was assessed based on two one-sided test procedures, in which the 90% confidence intervals (CI) were calculated. The 80–125% limits were used as the acceptance criteria for BE.

## 3. Results

### 3.1. Single Phase In Vitro Dissolution Test

**Figure 2:**
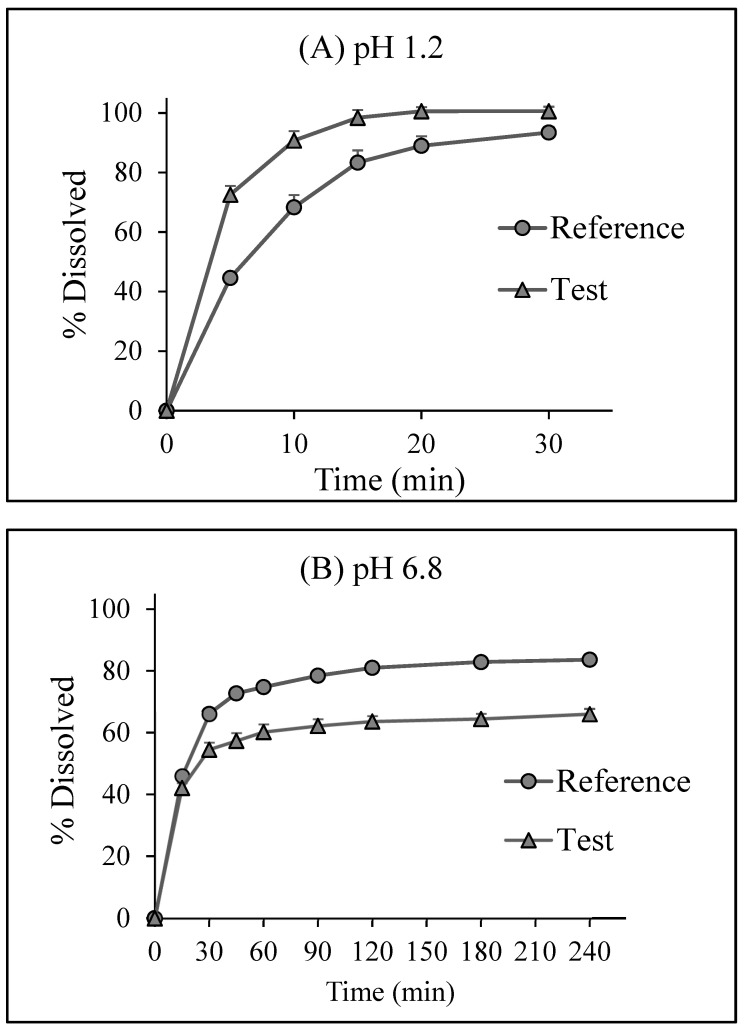
Dissolution profiles of 200 mg lamotrigine (LTG) reference and test tablets in the single phase dissolution test under (**A**) Sink condition (pH 1.2 hydrochloric acid, 900 mL) and (**B**) Non-sink condition (pH 6.8 phosphate buffer, 900 mL). Data were obtained using USP Apparatus II with a rotation speed of 50 rpm at 37 ± 0.5 °C (mean ± standard deviation (SD); *n* = 6).

Single-phase dissolution profiles in pH 1.2 and pH 6.8 dissolution media are presented in Figure 2. The dissolution of the products was rapid at pH 1.2 (>80% in 15 min) due to the sink condition in 900 mL of pH 1.2 HCl, which is the recommended medium for LTG tablets using the FDA dissolution database [41]. In a pH 1.2 medium, the drug release was 83% and 98% after 15 min for the reference and test, respectively (Figure 2). By contrast, the solubility of LTG at pH 6.8 was low (0.136 mg/mL), resulting in a non-sink condition in 900 mL of a pH 6.8 phosphate buffer. The drug release was much slower at pH 6.8 (75% and 60% in 1 h for the reference and test, respectively) compared to pH 1.2 (Figure 2). The test and reference dissolution profiles differed at pH 6.8 (*f*_2_ = 41). The *C_S_* values, calculated *D_O_*, and relative sink conditions (*C_S_*/*C_D_*) at 37 °C are presented in Table 1. The sink condition is considered to be provided for *C_S_*/*C_D_* values greater than three [42]. Therefore, the sink condition was provided in a pH 1.2 HCl medium. *D_O_* was less than one at pH 1.2; however, the value was greater than one (5.9) at pH 6.8 medium.

### 3.2. Biphasic In Vitro Dissolution Test

**Figure 3:**
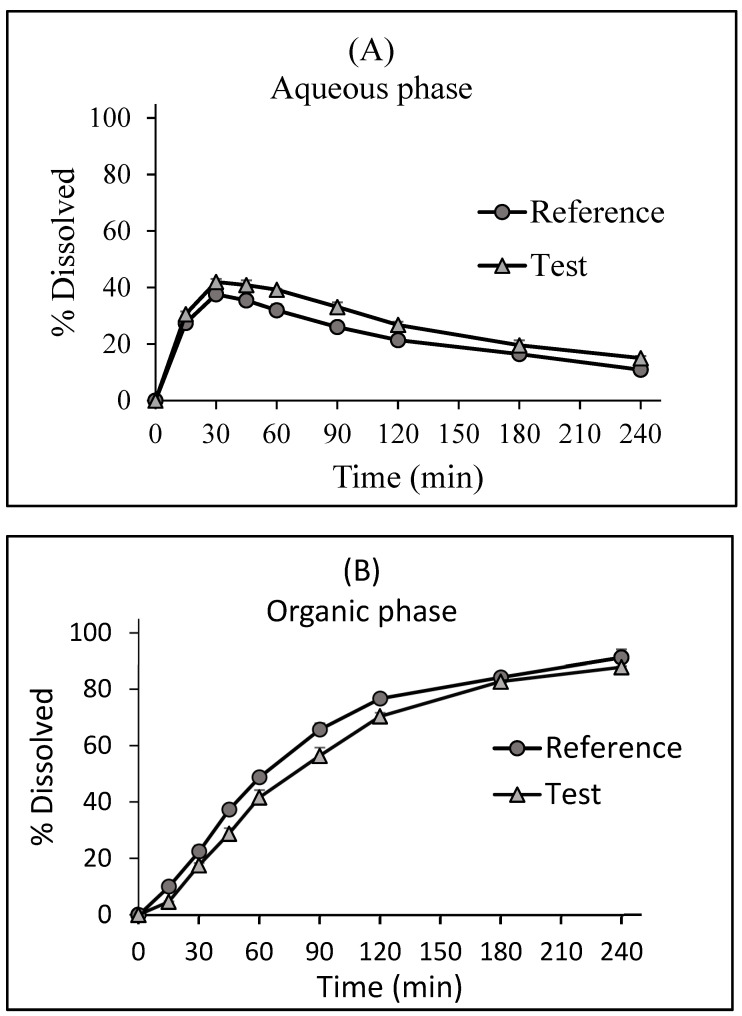

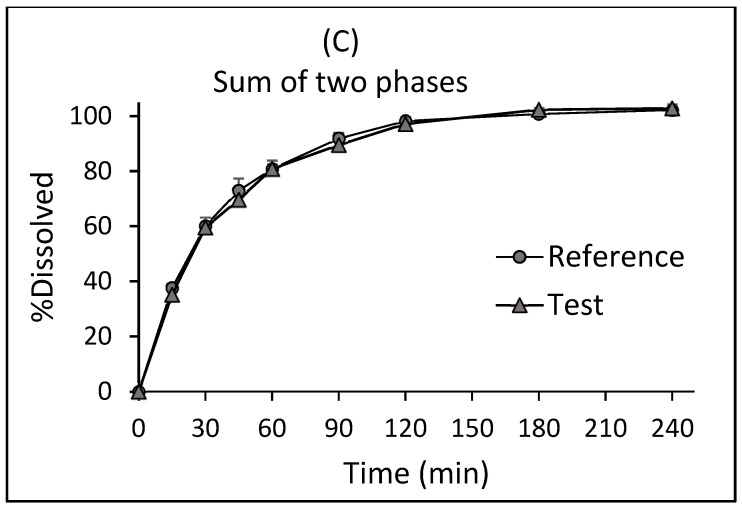
Dissolution profiles of 200 mg of the LTG reference and test tablets from the (**A**) aqueous (pH 6.8 phosphate buffer), (**B**) organic (octanol) phases, and (**C**) sum of two phases (total) in the biphasic dissolution test. Data were obtained using the paddle-modified USP Apparatus II with a rotation speed of 50 rpm and 37 ± 0.5 °C (mean ± SD; *n* = 3).

Biphasic dissolution profiles in the buffer, octanol, and the sum of two phases are presented in Figure 3. The test and reference dissolution profiles were similar in aqueous (*f*_2_ = 63.7) and organic phases (*f*_2_ = 59.4). In addition, the *k_p_* values for the reference and test were 0.66 and 0.63 h^−1^, respectively, indicating similar partitioning into the octanol from both products. The biphasic dissolution study demonstrated the highest percentage of LTG dissolved by the products at 30 min and the relative slowdown between 30 min–4 h in the buffer phase. The partitioning of LTG into the organic phase was relatively slow after 15 min, accelerated between 15 min and 2 h, and continued to increase slowly between 2 and 4 h for the reference and test products.

### 3.3. Relation between In Vivo Absorption and In Vitro Partitioning Data

**Figure 4:**
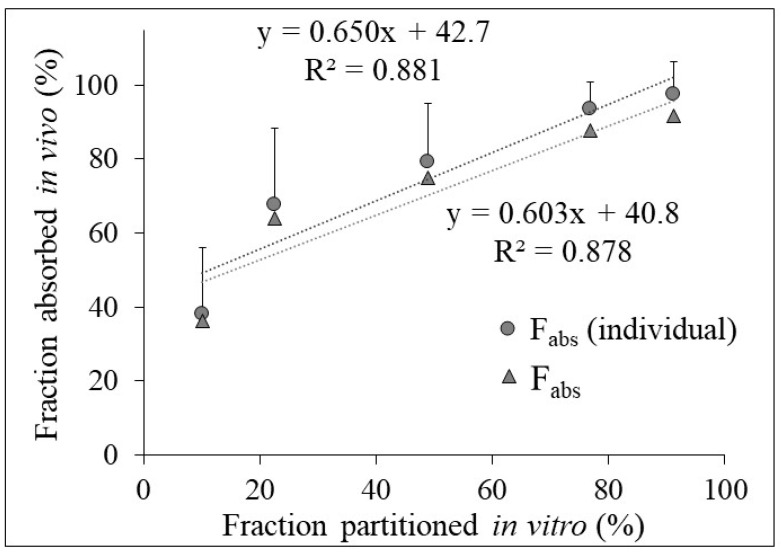
Correlation between LTG’s fraction of the dose absorbed (*F_abs_*) and the fraction of LTG partitioned into the organic phase between 0 and 4 h for the reference. (The circles represent the individual plasma data; the triangles represent the mean plasma data in healthy volunteers).

The correlation between the calculated LTG’s *F_abs_* (%) and the fraction of LTG partitioned into octanol (%) for the reference is presented in Figure 4. An adequate correlation was captured between in vitro partitioning into the organic phase and in vivo *F_abs_* values calculated from individual and mean plasma drug concentration data for the reference (r^2^ = 0.881 and 0.878, respectively). The calculated mean *k_a_* value of the test was 1.33 ± 0.61 h^−1^, while the observed mean *k_a_* value of the reference was 2.26 ± 1.09 h^−1^.

### 3.4. Prediction of In Vivo Plasma Profiles from the Biphasic In Vitro Data

**Figure 5:**
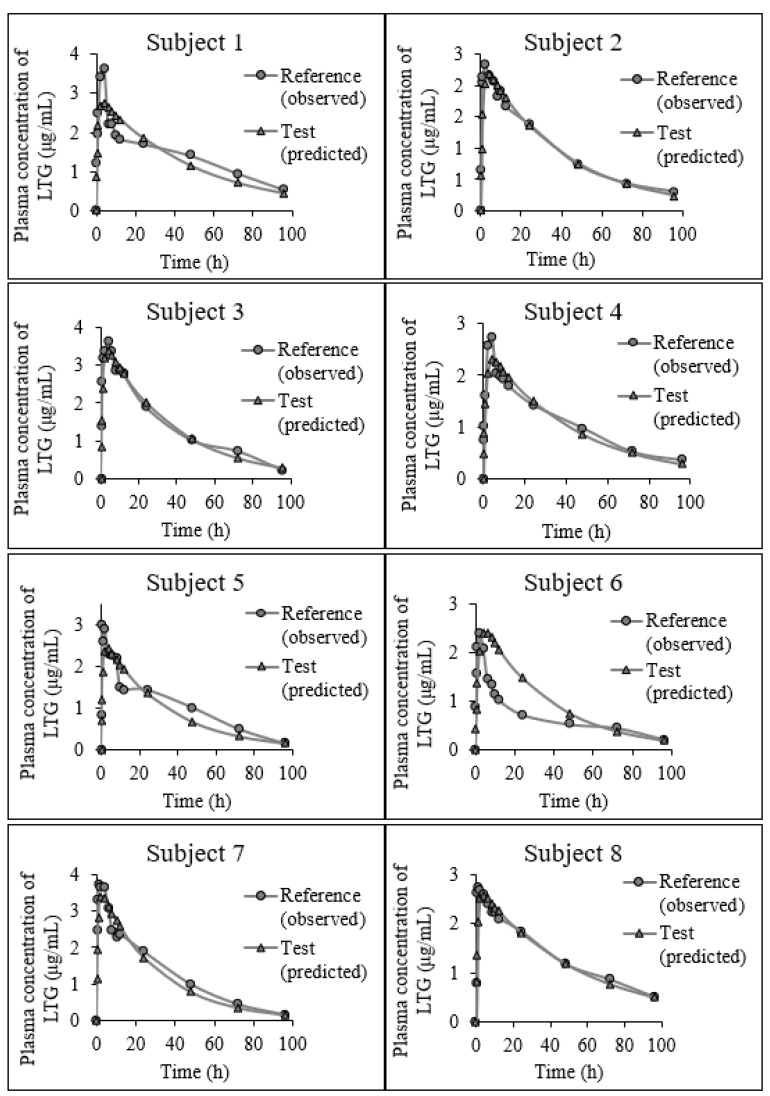

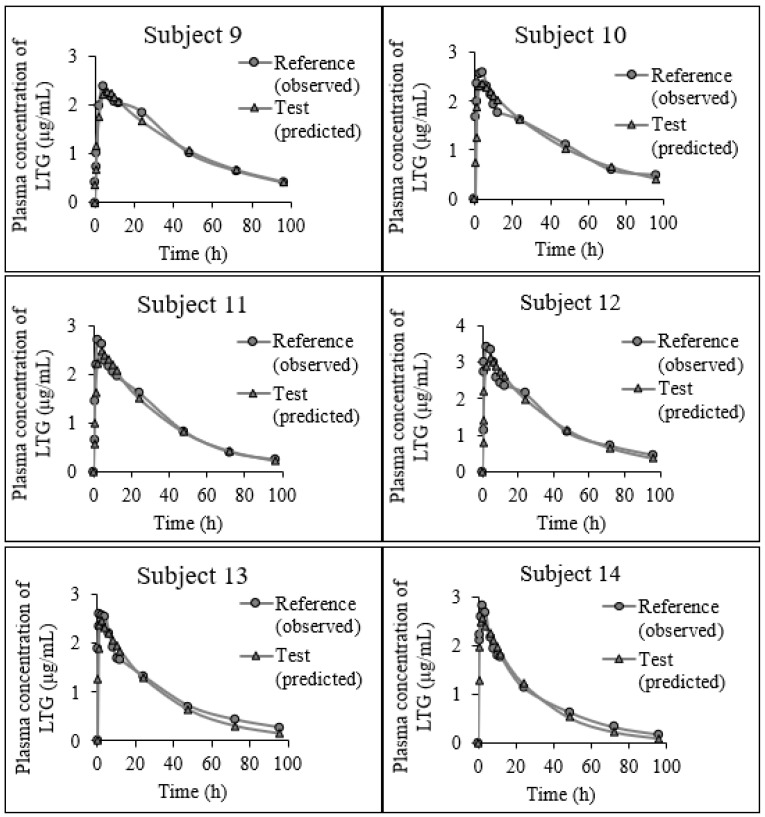
The plasma concentration–time profiles of LTG in 14 healthy volunteers (The circles represent the observed plasma profiles for the reference; the triangles represent the predicted plasma profiles for the test).

**Figure 6:**
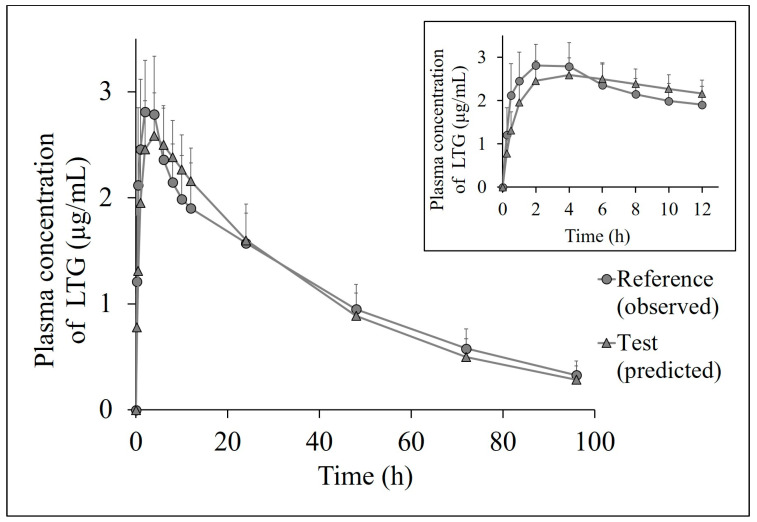
The mean plasma concentration–time profiles of LTG in healthy volunteers (The circles represent the observed plasma profile for the reference; the triangles represent the predicted plasma profile for the test) Data are presented as the mean ± SD, *n* = 14.

The predicted individual and mean plasma profiles of LTG for the test product compared to the observed plasma profiles for the reference in healthy volunteers are presented in Figure 5 and Figure 6, respectively.

The *AUC*, *C_max_*, and time to reach *C_max_* (*t_max_*) values are shown in Table 2. The generic product was found to be bioequivalent to the original product in terms of 80–125% BE criteria with a 90% Cl.

## 4. Discussion

The present study investigated the in vivo relevance of a biphasic dissolution test for LTG’s reference and test IR tablets. Plasma profiles for the test product were predicted in humans from biphasic dissolution data and compared with the in vivo results of the reference. LTG (MW: 256), a BCS Class II drug, was selected based on its high permeability and poor solubility in this study. Furthermore, previously reported human plasma data for the reference product of LTG were available to correlate in vitro and in vivo data [37].

LTG is a weak base with a pK_a_ of 5.7 and a log P of 1.93 [43,44]. Due to the basic structure, it demonstrates a pH-dependent solubility (3.63 and 0.136 mg/mL at pH 1.2 and 6.8, respectively). The physicochemical properties of drugs are among the critical factors affecting their GI absorption [45]. Accordingly, the high unionized fractions of LTG at jejunal (~pH 6.0) and ileal (~pH 7.4) pHs could explain its rapid absorption after the oral dose (*t_max_* = 1–3 h [46]). Thus, the lipophilic structure, basic characteristics, and pH-dependent profile of the unionized fraction of LTG revealed its high intestinal permeability. In addition, the *D_o_* of LTG (0.22 (pH 1.2) and 5.9 (pH 6.8)) indicate complete dissolution in the fasting stomach (pH 1.4–2.1; [47]). However, LTG seems to be in a supersaturated state in the upper intestine (pH 4.4–6.6; [47]), where it can be rapidly and extensively absorbed from its primary site of absorption before precipitating at high pH values of the distal regions of the small intestine. It has been reported that these basic drugs can be well absorbed in this supersaturated state from the intestinal mucosa [48,49].

When considered together with the results of the dissolution study at pH 1.2, it appears that more than 85% of the highest dose (200 mg) of LTG is released in the stomach within 10–20 min and is then rapidly absorbed in the upper intestine. However, conventional single-phase dissolution tests are unlikely to estimate the in vivo behavior of oral formulations of LTG. It highlights the need for a dissolution test to simulate in vivo behavior and be used in developing and evaluating oral formulations containing poorly water-soluble drugs. It was demonstrated that the biphasic dissolution test can accurately reflect in vivo drug release, solubility, and absorption for the evaluation of IR formulations of LTG in the present study. In the biphasic dissolution test, the octanol phase reflected the intestinal absorption of LTG. It has also been widely used in other biphasic dissolution studies in the literature since octanol can mimic biological membranes [50,51,52,53]. LTG has a suitable solubility in octanol (4.14 mg/mL) and a high affinity to octanol, making this drug a good candidate for the biphasic dissolution test. Indeed, the sink condition was maintained in 200 mL of the octanol phase for LTG in this study. Generally, a volume of 300–500 mL is suggested to be of in vivo relevance [3], complying with the present study. Consequently, the in vitro similarity of the formulations was verified in both phases.

The partitioning of LTG from the buffer into octanol from the reference and test was rapid between 15 min and 2 h due to rapid drug release from dosage forms, whereas the slow partitioning after 2 h was related to the equilibrium of two phases rather than drug release, which is consistent with the previous findings in other studies [52,54]. The partitioning of the drug from the buffer medium into the organic solvent is considered to be an important parameter for optimizing biphasic tests, reflecting the absorption [55,56]. Since the relationship between *k_p_* (in vitro) and *k_a_* (in vivo) is important, the proximity of the two rate constants, *k_p_*, and *k_a_*, can be used to develop a physiologically meaningful in vitro test. This approach recognizes that the drug exhibits high absorption in vivo with first-order absorption kinetics [24]. In the case of the present biphasic test, the in vivo *k_a_* (2.26 h^−1^) value was approximately three times higher than the calculated in vitro *k_p_* value for the reference. Therefore, the in vitro system was considered to be close to in vivo conditions and appropriate for evaluating the IR tablet formulations of LTG. Moreover, similar drug partitioning into the octanol phase was obtained for the reference and test formulations, suggesting the similarity of the formulations.

The present study suggests that the target release percentage of LTG in the organic phase is approximately 70–75% within 2 h, 80% within 3 h, and 90% within 4 h to achieve good in vivo performance to develop bioequivalent IR formulations of LTG. The partitioning of LTG into the organic phase within 4 h is also consistent with the finding that LTG is rapidly and completely absorbed from the GI tract, considering that the transit time from the small intestine is approximately 4 h in the fasting state [46,57].

It is essential to establish a correlation between in vitro and in vivo and to predict the in vivo plasma profiles through compartmental models to evaluate the in vivo significance of biphasic dissolution tests. Although IVIVCs have been described for controlled release formulations in the guidelines, good correlations between in vitro data from biphasic dissolution tests and in vivo human data have been found for IR formulations of BCS Class II drugs (celecoxib, deferasirox, racecadotril, ritonavir, and fenofibrate) over the last decade [10,28,51,54,55]. Among these, level A IVIVC was only established for deferasirox, ritonavir, and fenofibrate [10,54,55]. In a recent study by Denninger et al., the commercial solid dosage forms of five drugs (aprepitant, celecoxib, itraconazole, nimodipine, and ritonavir) were investigated in a newly developed small-scale system consisting of 50 mL of organic and aqueous phases [58]. The investigators established a level A IVIVC between the drugs’ profiles in the organic phase and human plasma. Then, they predicted each drug’s in vivo plasma profiles via compartmental modeling, suggesting that the in vitro system could predict in vivo profiles [58].

In the present study, LTG’s *F_abs_* were calculated using deconvolution. The Wagner–Nelson method was used to obtain *F_abs_* values from the plasma concentration–time curves for the reference product of LTG. The mean *F_abs_* value calculated separately from each plasma profile from 14 healthy volunteers was close to that calculated from mean data. There is a good correlation (r^2^ = 0.999) between the time-dependent *F_abs_* values calculated using these two methods, and the difference between these values is in the range of 4.6–6.1%. Therefore, it is concluded that the mean and separate plasma profiles can be used to establish the point-to-point correlation between the partition data and *F_abs_* values, as pointed out by others [10]. However, each plasma profile of the reference was evaluated separately for the correlation to predict the time–plasma concentration profiles of the test for each healthy volunteer in the present study. The correlation between the in vivo *F_abs_* and in vitro fraction of LTG partitioned into octanol was used for the predictions since the dissolution data from the octanol phase are generally used to establish IVIVC for solid dosage forms [10,28,54].

The correlation between in vivo and in vitro data was successfully applied to predict human plasma profiles for the test product using the compartmental model. The *k_a_* value of the reference (*k_a_* = 2.26 ± 1.09 h^−1^) was 1.7 times higher than that of the test product (*k_a_* = 1.33 ± 0.61 h^−1^), calculated using the Wagner–Nelson method. Similarly, the observed *t_max_* for the reference was 2.5 h, whereas *t_max_* was estimated to be 3.7 h for the test. The assessment of BE for the test versus reference ensured that the generic drug does not differ from the reference drug, which was in good agreement with the knowledge that the two formulations are bioequivalent.

Overall, biphasic in vitro dissolution testing seems to be a convenient procedure to estimate the performance of drug products containing poorly water-soluble active pharmaceuticals, providing potential in vitro-in vivo relationships. The present study found a high correlation between in vitro partitioning and absorption data of LTG’s IR formulation for the first time. The plasma profiles were estimated based on in vitro partition and drug disposition. It was concluded that the described biphasic test is likely to provide a discriminative and predictive power for the IR formulations of LTG. Consequently, this approach may result in considerable time and cost savings when developing oral formulations containing BCS Class II drugs.

## Figures and Tables

**Table 1:** Saturation solubility (*C_S_*) values, relative sink conditions (*C_S_*/*C_D_*), and dose numbers (*D_O_*) of lamotrigine (LTG) in hydrochloric acid (pH = 1.2) and a phosphate buffer (pH = 6.8) at 37 °C.

Medium	*C_S_* ^1^ (mg/mL)	*C_S_*/*C_D_* ^2^	*D_O_* ^3^
pH 1.2 hydrochloric acid	3.63 ± 0.01	16.3	0.22
pH 6.8 phosphate buffer	0.136 ± 0.001	0.611	5.9

^1^ *C_S_* is the saturation solubility of LTG; ^2^ *C_D_* is the theoretical concentration of the drug, assuming complete dissolution of 200 mg LTG tablet in 900 mL dissolution medium; ^3^ *D_o_* is the dose number.

**Table 2:** Bioavailability (BA) criteria for test and reference products.

	Test		Reference		
BA Criteria	Mean ± SD ^1^	CV% ^2^	Mean ± SD	CV%	BE Limits for 90 % CI ^3^
*AUC* (µg/mL.h) ^4^	119 ± 24.9	21.0	123 ± 14.3 *	11.6	85.9–107
*C_max_* (µg/mL) ^5^	2.61 ± 0.39	15.1	2.90 ± 0.26 *	8.96	82.7–97.6
*t_max_* (h) ^6^	3.7 ± 1.1	28.8	2.4 ± 0.7 *	29.2	

^1^ standard deviation; ^2^ coefficient of variation; ^3^ confidence interval; ^4^ area under the LTG concentration–time profile calculated over the 0 to infinity time interval; ^5^ peak plasma concentration of LTG; ^6^ time to reach *C_max_*, * Data are obtained from Incecayir et al. 2007 [37].

## Data Availability

The data presented in this study are available in this article.
